# Hydrocortisone Therapy in Post-Stroke Management of Persistent Hiccups: A Case Report

**DOI:** 10.7759/cureus.56800

**Published:** 2024-03-23

**Authors:** Katia Latifa, Mariam Ibrahim Al Ali, Hafsah Areen, Liza Thomas, Uzma Sabahat

**Affiliations:** 1 Internal Medicine, Dubai Health, Dubai, ARE; 2 Internal Medicine, Mohammed bin Rashid University of Medicine and Health Science, Dubai, ARE

**Keywords:** medulla ischaemia, intractable hiccups, persistent hiccups, dexamethasone, hiccups treatment, posterior circulation stroke, hydrocortisone, hiccups, steroids

## Abstract

Hiccups, also known as singultus, are involuntary spasms of the diaphragm muscle followed by laryngeal closure involving a reflex arc. It is a relatively common phenomenon, usually transient and self-limiting. However, in medical settings, it could be much more serious and is often a sign of underlying pathology.

When hiccups last for over 48 hours, they are referred to as persistent hiccups, and if they persist for more than a month, they are known as intractable hiccups. Current pharmacologic treatment of persistent or intractable hiccups mainly includes antidopaminergic drugs, which specifically antagonize the dopamine D2 receptor.

Here, we present the case of a 54-year-old gentleman who was admitted under our care with a posterior circulation stroke specifically affecting the medulla. He was symptomatic with severe, persistent hiccups interfering with sleep and oral intake and unresponsive to all standard medications. After nearly two weeks, a trial of hydrocortisone was given, to which he responded dramatically.

To the best of our knowledge, this is the only case of hiccups that has been successfully treated with hydrocortisone. The remarkable improvement seen in our patient when treated with hydrocortisone suggests hydrocortisone could be a useful agent in post-stroke hiccups that are unresponsive to traditional treatment for hiccups.

## Introduction

Hiccups, also known as singultus, are involuntary contractions of the diaphragm muscle followed by laryngeal closure [1]. They arise from a state of hyperexcitability in the reflex arc, involving three main components: firstly, the afferent limb, responsible for receiving signals from either the central nervous system or irritated peripheral nerves, secondly, central brain processing; and thirdly, the efferent limb, where the brain sends signals through nerves to the diaphragm and respiratory muscles, triggering the characteristic hiccup response [2]. Neurotransmitters such as dopamine (D) and gamma-aminobutyric acid (GABA) are implicated in this phenomenon [3].

Most research has focused on persistent and intractable hiccups that are categorized based on their duration: acute hiccups last less than 48 hours, persistent hiccups persist for over 2 days, and intractable hiccups last for more than a month [4]. Several typical human behaviors and conditions can cause arc reflex dysfunction, such as toxic-metabolic causes (for example hyponatremia, hypokalemia, hypocalcemia, hyperglycemia, uremia), chronic alcoholism, esophageal and gastric tumors, pleural involvement, mediastinal or diaphragmatic lesions, and intracranial lesions [4]. One of the common causes of intractable or persistent hiccups is posterior circulation ischemic stroke, particularly in the acute phase [5].

Currently, antidopaminergic (dopamine D2 receptor antagonists) and GABA-modulating medications such as baclofen, haloperidol, gabapentin, and chlorpromazine are used as pharmacological management for persistent or intractable hiccups [6]. In this case report, we describe a patient diagnosed with post-stroke persistent hiccups who showed remarkable improvement after being treated with hydrocortisone, despite prior unsuccessful attempts with typical hiccup medications.

## Case presentation

A 54-year-old man with a past medical history of hypertension (not compliant with medication) and dyslipidemia (not on medication) presented to our emergency department with symptoms of right-sided weakness, headache, dizziness, slurred speech, blurred vision, difficulty swallowing, and hiccups. Upon arrival at the emergency department, the patient had a GCS of 15, was hypertensive with a blood pressure of 167/78 mmHg, and was otherwise vitally stable. His examination at presentation was significant for reduced power over the right upper and lower limb graded as 3/5, with normal tone and reflexes. Dysphagia on solids and liquids was also observed. No aphasia, dysarthria, sensory deficits, or facial asymmetry were noted. Due to his significant dizziness and nausea, coordination, extraocular muscles, and nystagmus could not be evaluated accurately. The patient had a similar episode a month ago with neck pain and left-sided weakness when he presented to a private clinic and was diagnosed with hypertension and started on a calcium channel blocker. A CT brain performed at presentation showed an occluded V2 segment above the C4 level, and V3 and V4 segments of the right vertebral artery were described as likely thrombotic, no acute infarct was detected, and no acute hemorrhage. As he was out of the window for tPA and not eligible for mechanical thrombolysis, the patient was started on single antiplatelet therapy, and an NG tube was inserted due to his dysphagia.

**Figure 1:**
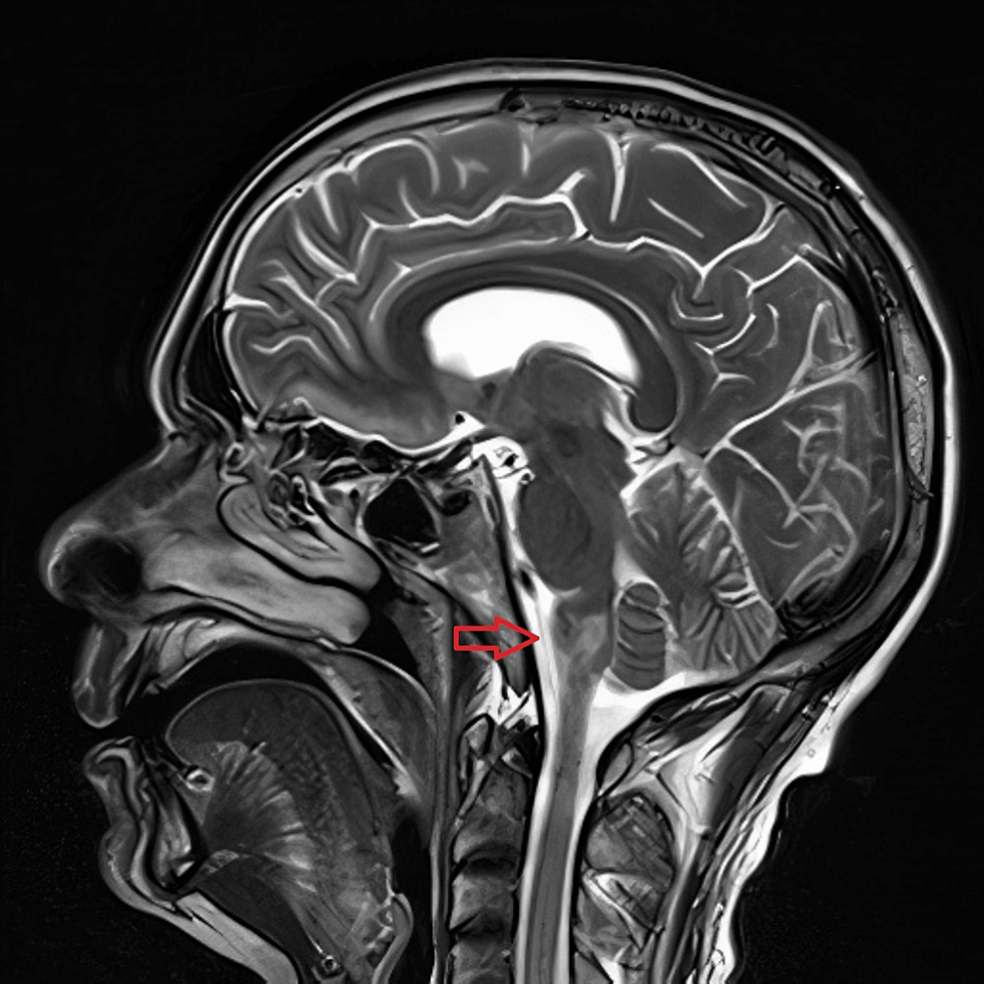
Sagittal T2-weighted brain MRI with posterior circulation infarct in the right medulla oblongata

**Figure 2:**
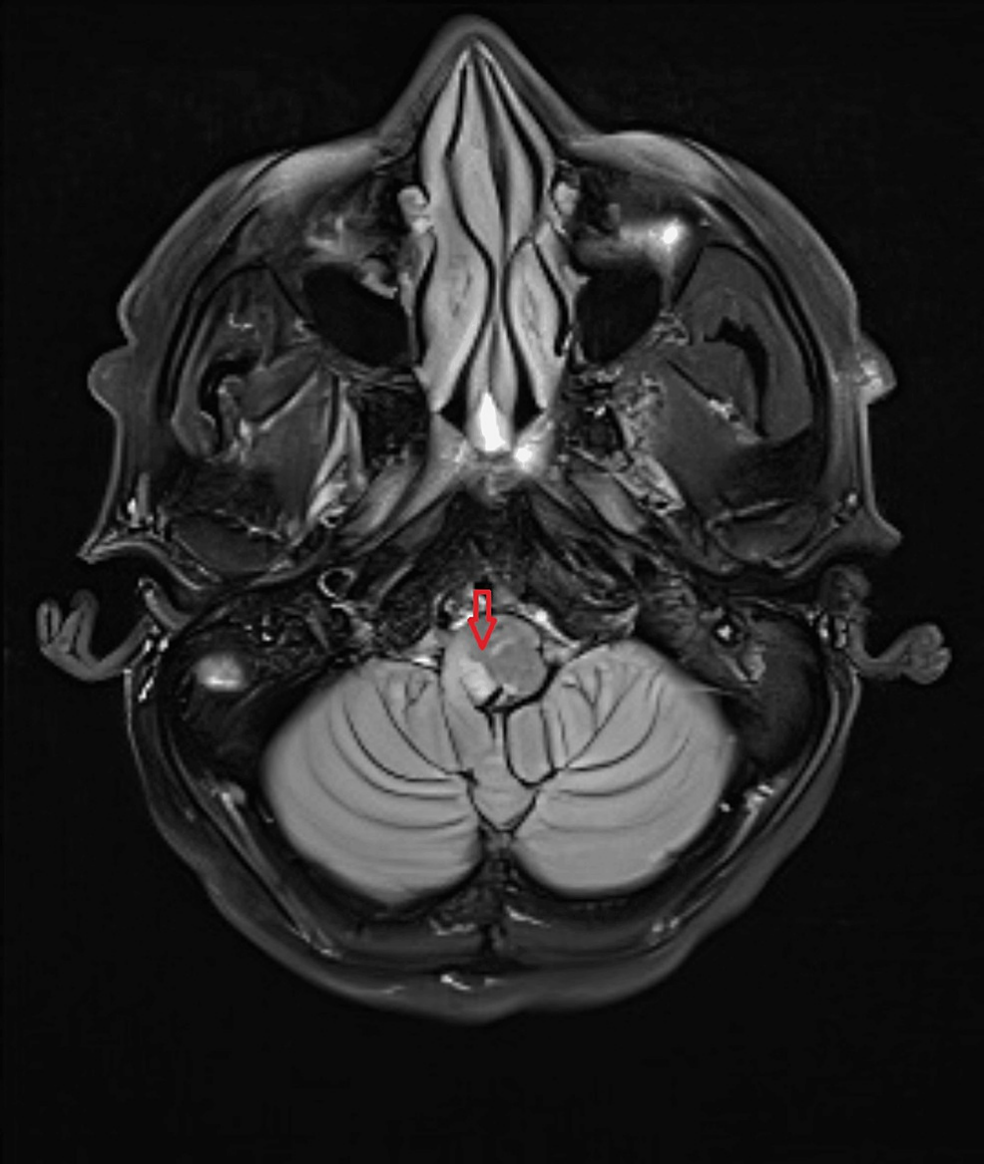
Axial brain MRI with posterior circulation infarct in the right medulla oblongata

**Figure 3:**
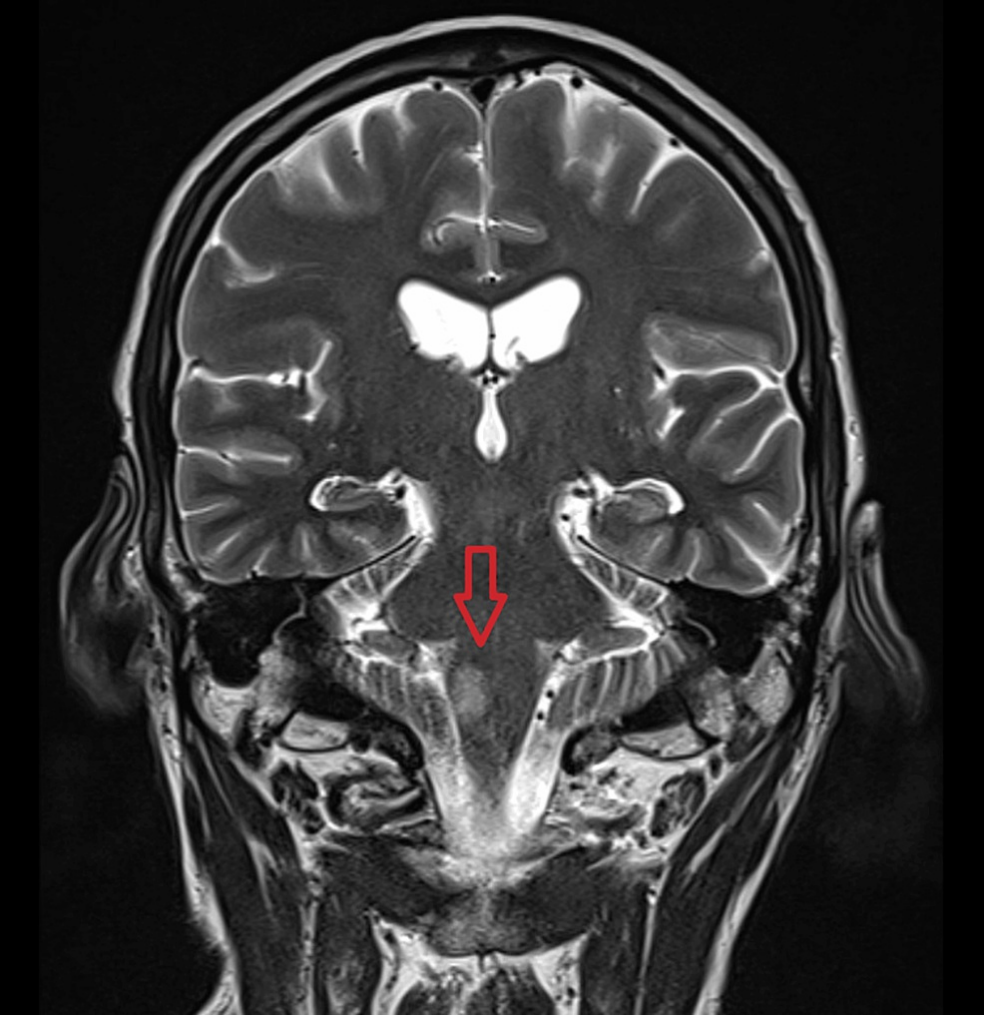
Coronal brain MRI with posterior circulation infarct in the right medulla oblongata

CT brain was done 24 hours after the onset of symptoms and showed no changes compared to the initial study. As an inpatient, he was managed with a calcium channel blocker and vasodilators PRN for his hypertension, aspirin, and atorvastatin, with early initiation of inpatient physiotherapy once the patient was stabilized. The patient’s primary concern was his symptomatic hiccups, which were persistent, lasting throughout the day, and severely disabling as the patient was unable to sleep at night, developed chest discomfort due to constant hiccups, and was unable to complete sentences when speaking. MRI showed features of subacute posterior circulation infarct in the right medulla oblongata (Figures 1-3).

**Figure 4:**
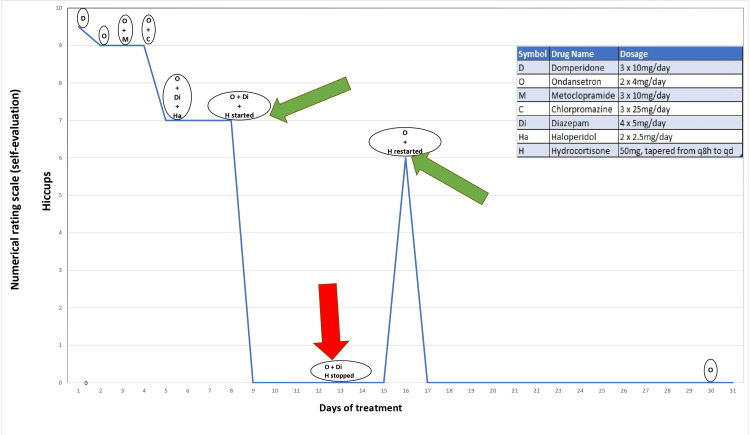
Severity of hiccups over the course of treatment: The graph illustrates the medication regimen doses and corresponding changes in the severity of hiccups

He required multiple medications, including domperidone, ondansetron, and metoclopramide. Metoclopramide was eventually stopped and replaced by chlorpromazine, but no significant improvement in hiccups was observed. Intramuscular haloperidol was also added later, along with diazepam and PRN procyclidine, but the patient remained distressed due to continuous hiccups. An attempt at removing the NG tube also showed no considerable difference. At this stage, the patient was started on IV hydrocortisone 50 mg TDS for 5 days along with continuing the ondansetron and diazepam, to minimize any edematous changes. Following this, the patient showed dramatic improvement in symptoms. However, the hiccups resumed after hydrocortisone was stopped after a 5-day course. Due to this, hydrocortisone was restarted, and the hiccups settled once again. Figure 4 shows how the severity of the hiccups changed over time during the treatment.

In terms of weakness, nausea, and dizziness, the patient's symptoms improved over the first few days of admission. Although his dysphagia and saliva pooling symptoms were gradually improving, he was kept on NG feeding because of repeated swallow assessments that were unsuccessful. The hiccups eventually settled after starting and tapering down hydrocortisone. He was doing well overall with well-controlled blood pressure, walking independently, and tolerating tube feeding. He was discharged with amlodipine/valsartan, ondansetron, aspirin, atorvastatin, betahistine (for 7-10 days), and a PPI with advice for a follow-up swallow study in 1-2 months.

## Discussion

To the best of our knowledge, there have been no previous cases reported with persistent hiccups that resolved with hydrocortisone or other corticosteroid use. As mentioned in the literature, the exact center for hiccups in the human brain is not well known; however, it appears that parts of the central nervous system (CNS) involved in the mechanism of hiccups are the upper spinal cord (C3-C5) and brainstem in the medulla oblongata near the respiratory centers, as well as the hypothalamus [7]. Other research suggests that the nucleus ambiguus plays a role in the mechanism of hiccups [8].

As mentioned, our patient had an initial presentation of right-sided weakness, headache, dizziness, slurred speech, blurred vision, difficulty swallowing, and hiccups, which were treated according to the stroke protocol. Further imaging studies such as CT brain were conducted, and based on the history provided with no previous such episodes, stroke was ruled to be the cause of the persistent hiccups. Treatment of hiccups lies within addressing the underlying cause; for instance, if the cause is gastroesophageal reflux disease (GERD), then treating it indirectly treats the hiccups. However, for other causes that are not directly treatable, considering treatments specifically targeting hiccups is the option to opt for.

As discussed in the literature and by reviewing a systematic review done in 2015, the line of treatment for intractable hiccups, i.e., hiccups that last longer than a month, is divided into non-pharmacological therapy and pharmacological therapy. This includes first-line treatment (baclofen, gabapentin, pregabalin), second-line treatment (metoclopramide, domperidone), and other choices (carbamazepine, valproate, phenytoin, nifedipine, and amitriptyline). It is important to mention that each medication has its own limitations and side effects [7] and needs to be tailored to the specific needs and preexisting conditions of each individual.

In our case, the patient was initially given domperidone, ondansetron, and metoclopramide, which improved some symptoms that he initially presented with; however, none of the classical or well-known treatment options was effective in subsiding the hiccups. An NG tube was inserted in an attempt to reduce the hiccups; however, it was not effective. It was noted in the literature that nerve block (phrenic nerve, vagal nerve) was one of the non-pharmacological options for such cases; however, it is extremely selective with a possibility of the individual suffering from pneumothorax [9]. Anesthesia consultation regarding elective nerve block was done, and it was contraindicated, and opting for other possible therapies was suggested.

Regarding the causes of hiccups, steroids, and to be more precise, dexamethasone, was found to be one of the hiccups-inducing agents. It was found to have a higher rate of causing hiccups in comparison to other corticosteroids. While the exact mechanism is not fully known, corticosteroids are thought to cause hiccups by lowering the synaptic transmission threshold in the midbrain and directly triggering the central part of the hiccup reflex arc [10].

A case series study done in 2012 states that the possibility of dexamethasone causing hiccups could be due to its unique chemical structure, affinity to hiccup receptors in the brain stem, its potency, and/or its dispersion in the body. Within this study, dexamethasone was initially used for the prevention of chemotherapy-induced nausea and vomiting; however, it was found to induce hiccups as a side effect. Hence, prednisolone was used as an alternative option, and the hiccups ceased. It was not determined if prednisolone does not induce hiccups or if it stops them [11].

Steroids are found to be effective in reducing inflammation and edema. Unfortunately, there has not yet been solid proof of the effectiveness of corticosteroid use in patients with stroke. However, a study summarizes that many physicians have used corticosteroids in patients with stroke [12].

In the case of our patient, due to the failure of all traditional pharmacological therapies, the suggestion of using corticosteroids (hydrocortisone) was made, as it has potential effects on reducing edema. IV hydrocortisone 50 mg TDS was started in week 2 of admission, and the patient no longer had hiccups, which in turn improved his sleep and diet. An attempt to stop hydrocortisone was made to assess responsiveness and the possibility of relapse. This attempt was unsuccessful as the patient’s symptoms relapsed, and hiccups started again.

Our recommendation is to conduct studies showing the possible effect of certain corticosteroids on patients with hiccups resistant to the traditional lines of treatment, especially for hiccups post-cerebrovascular events.

## Conclusions

As discussed, persistent or intractable hiccups can manifest in various conditions, including, as seen in our case, post-posterior circulation stroke. Severe hiccups, such as those experienced by our patients, can be highly debilitating, sometimes more so than the primary neurological symptoms.

When all medical modalities have been exhausted, surgical modalities become the next course of action. Hydrocortisone has never been attempted for this indication before, and it could represent a novel and promising medication, given its low side effect profile. However, further reports and studies are necessary to explore its mechanism of action and confirm its beneficial role in managing intractable hiccups.
